# The Use of Antimicrobial and Antiviral Drugs in Alzheimer’s Disease

**DOI:** 10.3390/ijms21144920

**Published:** 2020-07-12

**Authors:** Umar H. Iqbal, Emma Zeng, Giulio M. Pasinetti

**Affiliations:** Department of Neurology, Icahn School of Medicine at Mount Sinai, New York, NY 10029, USA; umar.iqbal@mssm.edu (U.H.I.); emma.zeng@mssm.edu (E.Z.)

**Keywords:** Alzheimer’s disease, amyloid-β, antimicrobial, antiviral, antimicrobial peptide

## Abstract

The aggregation and accumulation of amyloid-β plaques and tau proteins in the brain have been central characteristics in the pathophysiology of Alzheimer’s disease (AD), making them the focus of most of the research exploring potential therapeutics for this neurodegenerative disease. With success in interventions aimed at depleting amyloid-β peptides being limited at best, a greater understanding of the physiological role of amyloid-β peptides is needed. The development of amyloid-β plaques has been determined to occur 10–20 years prior to AD symptom manifestation, hence earlier interventions might be necessary to address presymptomatic AD. Furthermore, recent studies have suggested that amyloid-β peptides may play a role in innate immunity as an antimicrobial peptide. These findings, coupled with the evidence of pathogens such as viruses and bacteria in AD brains, suggests that the buildup of amyloid-β plaques could be a response to the presence of viruses and bacteria. This has led to the foundation of the antimicrobial hypothesis for AD. The present review will highlight the current understanding of amyloid-β, and the role of bacteria and viruses in AD, and will also explore the therapeutic potential of antimicrobial and antiviral drugs in Alzheimer’s disease.

## 1. Alzheimer’s Epidemiology

Alzheimer’s disease (AD) is a progressive neurological disorder that accounts for the greatest number of dementia cases. As of 2019, 5.8 million people were living with AD, with its prevalence predicted to increase to 13.8 million by 2050 [1]. The vast majority of cases are concentrated in ages over 65, impacting 10% of people in this age group. In addition, the economic toll of AD on the United States economy is significant, estimated to be roughly USD 290 billion in 2019 [1]. As the number of cases is only expected to rise over the coming decades, research in this field is critical in order to understand the pathology of this disease, as well as potential therapeutics.

## 2. Timeline/Characterization of Alzheimer’s Disease

The understanding and characterization of AD can be traced back over 100 years to Alois Alzheimer, from whom the disease takes its name. After completing an autopsy of a patient with progressive dementia, Alzheimer noticed a severe amount of cortical degeneration and an accumulation of protein deposits, specifically extraneuronal plaques and intraneural tangles [2]. By 1991, the buildup of extraneuronal amyloid-β (Aβ) plaques became the hallmark trait of the pathogenesis of Alzheimer’s disease [3], initiating the development of the amyloid cascade hypothesis [4]. In parallel to Aβ plaques formation, the accumulation of other naturally unfolded proteins is central to AD and other cerebral proteopathies [5]. The intracellular aggregation of tau proteins in the form of neurofibrillary tangles (NFTs) is also an essential trait in the pathogenesis of Alzheimer’s disease [3,6]. A recent 2020 study found that neuroinflammation could play a role in the aggregation of tau, as DNA extracted from various bacterial species promoted tau misfolding [7]. Whereas Aβ plaques are more critical to AD pathogenesis, the tau protein appears to be more responsible for subsequent cognitive impairment and dementia symptoms associated with AD [8]. Indeed, tau hyperphosphorylation and NFT levels are closely correlated with cognition, and exhibit potential as therapeutic targets for AD treatment [9]. Furthermore, tau protein production has been shown to have a positive correlation with the production of Aβ plaques [10], with the formation and lack of clearance of Aβ plaques also being proposed to induce tau protein formation into NFTs [11]. Coupling this with the bi-directional relationship between Aβ plaques and neuroinflammation [12] would cement Aβ’s key role in driving AD pathology.

### 2.1. Amyloid-β Generation

**Figure 1:**
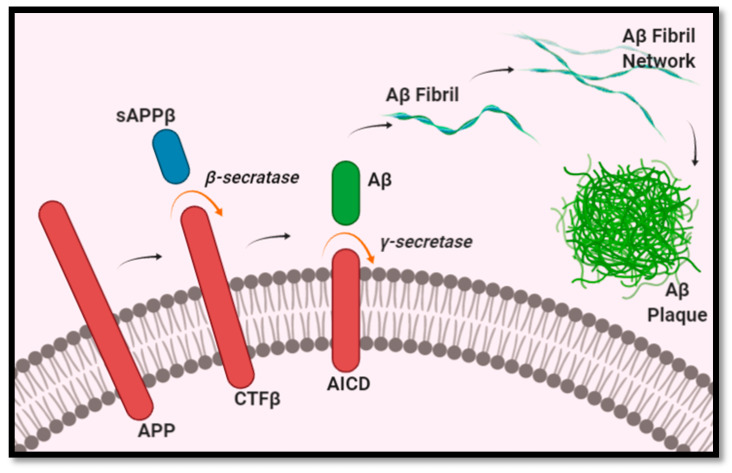
Generation of Aβ plaques.

Aβ formation begins with the breakdown of the amyloid precursor proteins (APP) embedded in the membranes of cells, such as neurons, as a type 1 transmembrane glycoprotein [13]. Aβ peptides are produced through a two-step cleavage process, in which APP is metabolized into smaller fragments. In the first step, APP is cleaved by β-secretase 1 into a membrane-bound CTFβ fragment (containing 99 amino acids) and an extracellular fragment sAPPβ. CTFβ is then further cleaved by γ-secretase to create the final Aβ peptide [13,14,15], as illustrated in Figure 1. The length of Aβ peptides is not fixed, and can consist of anywhere between 37 and 49 amino acids, depending on where the cleavage was done by β-secretase 1 and γ-secretase [16,17]. The most abundant length is Aβ_1–40_, representing approximately 80–90% of Aβ peptides, whereas the least soluble of the Aβ peptides, Aβ_1–42_, represents roughly 5–10% [13,15]. As a greater number of Aβ peptides form, they begin to aggregate into oligomers, which then form fibrils, and eventually the insoluble plaques characteristic of AD [13]. Of the different isoforms of Aβ peptides, Aβ_1–40_ and Aβ_1–42_ are the most common in plaques. Regarding the comparative role of Aβ_1–40_ and Aβ_1–42_ peptides in the pathogenesis of AD, Aβ_1–42_ peptides have been found in higher concentrations in AD. Furthermore, Aβ_1–42_ peptides have been found to be more prone to forming insoluble amyloid fibrils than Aβ_1–40_ [18]. This is further supported by a study using transgenic mice that expressed either Aβ_1–40_ (BRI- Aβ40) or Aβ_1–42_ (BRI- Aβ42A). The authors found that the mice that selectively expressed Aβ_1–40_ did not develop AD pathology at any age. However, the same did not hold true for BRI- Aβ42A mice, which had developed Aβ deposits [19]. In addition, another study found Aβ_1–42_ peptides to promote Aβ plaques formation, and Aβ_1–40_ to decrease Aβ deposition [20]. These findings would indicate the key role Aβ_1–42_ peptides play in the pathogenesis of AD.

### 2.2. Current Interventions and Limitations

Even with the tremendous effort that has been put into developing potential therapies for AD over the past few decades, there has been little success in reaching an effective therapy, with no new drug being approved in over a decade. While cholinesterase inhibitors and memantine are FDA-approved drugs for AD, and do address some of its symptoms, they lack the ability to attenuate disease progression. Over the past 20 years, a majority of the therapies have been based on the amyloid cascade hypothesis, and hence have focused on depleting Aβ peptides. Theses therapies often aim to inhibit γ-secretase or β-secretase activity in order to limit Aβ peptide production. Therapies that use such methods have, however, seen an increase in the rate of infection during clinical studies, with one study seeing 6% of participants develop meningoencephalitis [21,22]. Tarenflurbil, for instance, had been administered clinically, after it was shown to modulate γ-secretase and increase production of the less toxic Aβ_1–38_ peptide, rather than Aβ_1–42_ peptide [23,24]. However, in addition to not showing any significant benefit in individuals with mild AD, participants in the treatment group experienced an increase in upper respiratory infections and dizziness compared to the placebo group [25]. Additionally, in the time of COVID-19 infection, respiratory-related side effects, such as the ones related to tarenflurbil, are of growing concern. The γ-secretase inhibitor Semagacestat has not only been associated with increased levels of infection, but also with a failure to provide any cognitive improvement in patients with probable AD [26]. Furthermore, when patients with mild to moderate AD were administered ELND005, a compound that inhibits Aβ fibrils and plaque formation, it was observed that higher doses of this treatment led to serious infection. This led to lower dosage recommendations for future trials [27,28]. Lastly, the B-site ABPP cleaving enzyme 1 (BACE1)-inhibitor E2609 has also been associated with oralabial herpes relapse [29,30]. A rise in infection occurring in tandem with the reduction of Aβ peptide production could indicate these peptides’ potential role in immune function.

Through these past clinical trials, it is evident that therapies largely based on the amyloid cascade hypothesis, which in turn aim to eradicate Aβ peptides in the brain, have historically been ineffective. These failures could imply that current approaches either intervene at a stage that is too late, or possess a therapeutic target that is not as relevant to disease progression [31]. This would make sense in the context of AD especially, as Aβ deposition occurs 10–20 years before the occurrence of clinical symptoms [32]. Therefore, these treatments that target Aβ peptides specifically may already be too late. To create a successful therapy, it may be necessary to consider intervention in the presymptomatic stage of the AD instead. To do so, it would be crucial to identify biomarkers for early identification of AD. In March of 2016, a meeting was convened in which international, interdisciplinary experts identified a list of biomarkers that could be used for identifying AD early on [33]. CSF levels of Aβ_1–42_ and Aβ_1–40_, and the ratio of Aβ_1–42_/Aβ_1–40_, were determined to be among the candidates [33,34]. In addition, plasma levels of the same biomarkers were determined to decrease in AD patients when compared to healthy subjects [35]. Other biomarkers to consider would include plasma levels of tau protein and neurofilament light [33]. Plasma levels of the latter have been shown to be able to detect neurodegeneration in presymptomatic AD [36]. Other highly sensitive methods that have shown promise in the early identification of AD include protein misfolding cyclic amplification (PMCA) and real-time quaking-induced conversion (RT-QuIC), to determine Aβ oligomers and tau protein levels in CSF [37,38]. Taking a more preventative approach to AD treatment, and understanding and addressing factors that contribute to the progression of AD, may be favorable for identifying therapeutic targets earlier in the disease. Based on evidence of the association between bacterial/viral infection and AD progression, the antimicrobial hypothesis suggests that Aβ peptides may be produced as a protective mechanism by the innate immune system, and act as an antimicrobial peptide (AMP) against foreign agents. If Aβ peptides do in fact play a beneficial role in immunity, then the aim of treatment should not be to eradicate the compound entirely. Rather, it should be to target the root cause of its over production, and reduce its deleterious effects and general neuroinflammation in AD.

## 3. Antimicrobial Protection Hypothesis

### 3.1. Role of Microorganisms and Viruses in AD and Aβ Generation

Neuroinflammation is inflammation within the brain or spinal cord due to infection, toxins or injury [39]. In the brain specifically, resident glial cells, such as microglia and astrocytes, along with endothelial cells and mast cells, all aid in defending the brain against foreign pathogens [40]. Microglia, the main immune effector cells of the central nervous system (CNS), are constantly surveying their environment for potential threats to the brain [41,42]. When an invading agent is detected, microglia change into an activated state, characterized by an enlarged soma and the production of inflammatory cytokines and chemokines [39]. Astrocytes also play a critical role in mediating neuroinflammation as they are responsible for many neuroprotective functions, such as maintaining blood brain barrier (BBB) integrity and buffering neurotransmitters [43]. Upon injury, astrocytes likewise undergo morphological changes, and exhibit increased reactivity and secretion of cytokines and chemokines [41]. While acute inflammatory responses are common to healthy individuals, chronic inflammation is damaging to the natural balance of pro- and anti-inflammatory signaling in the brain, and can lead to the development and progression of neurodegenerative diseases like AD [39].

Over the years, there has been increasing evidence of neuroinflammation’s role in AD. In addition to Aβ plaques and NFTs, markers of sustained inflammation and microglial activation have repeatedly been found in AD brain samples [39]. The cytokines interleukin 1 and interleukin 6 are especially elevated [44]. One source of the neuroinflammation in AD patients could be the response to invading microorganisms and viruses. In fact, researchers have found evidence pointing to the presence of pathogens, such as viruses, bacteria and fungi, in AD brains [45,46,47,48,49,50]. This notion draws parallels with the measles virus, which can lead to the development of the neurological disease known as subacute sclerosing panencephalitis [51]. These findings include the identification of viral [52] and bacterial DNA in post-mortem brain samples, and the detection of pathogens [53] and/or their respective antibodies [54] in the serum or cerebrospinal fluid of patients. Furthermore, detection of lipopolysaccharide is commonly used by researchers to measure the presence of Gram-negative bacteria, like *P. gingivalis* specifically, as it is found in their cell walls and can stimulate an inflammatory response in the immune cells [42]. Herpes simplex virus-1 (HSV-1) [52,55,56,57,58] was the first pathogen found to be present in AD brain samples [59], and it thereafter became the most widely-researched pathogen regarding the linkage between viral infection and AD. Since then, other viruses have been identified in leading to the progression of AD, including human cytomegalovirus [54] and Epstein Barr Virus [60]. A recent 2018 study found that, in addition to HSV-1, herpesvirus types HHV-6 [46,47] and HHV-7 were highly present in AD patients [61]. In this study, by Readhead et al., HHV-6 and HHV-7 were also observed to be involved in regulatory processes critical to characteristic features of the disease [61].

Bacterial infection has likewise been associated with the progression of AD. The presence of bacteria in the brain has been determined in previous studies, suggesting the presence of a brain microbiome [62,63,64]. Even though bacterial presence has been seen in the brains of healthy individuals, tissue samples from AD brains have greater levels of bacterial species [64], indicating a greater level of infiltration. *Chlamydia pneumoniae* is the most widely-studied bacteria regarding association to AD [25,49]. A clinical investigation, made up of a healthy control group and an AD group, detected *C. pneumoniae* in 90% of the AD patients, whereas the control group were all negative [53]. *Escherichia coli*, likewise identified in AD brains [65], has been found to be capable of synthesizing extracellular amyloid [66]. Stains such as *Borrelia burgdorferi* [67], spirochetes [48], and *Porphyromonas gingivalis*, a pathogen commonly linked to chronic periodontitis, have also been identified in AD brain samples [68]. 

Interestingly, fungal infection from species primarily associated with periodontal disease has recently been suggested to be involved in AD progression. Researchers in a 2014 study detected multiple fungal species in AD brain samples, including *Saccharomyces cerevisiae*, *Malassezia globosa*, *Malassezia restricta*, *Penicillium* and *Phoma* [69]. Pisa et al. have since followed up on this initial discovery by analyzing the presence of these species between brain regions [70]. With any of these studies that have been conducted, however, it is important to recognize the technical limitations that arise when studying microorganisms and neurodegenerative disease. Many of these studies are limited to the use of post-mortem brain samples, and thus present the risk of contamination due to death or the passage of microbes from other areas of the body, such as the gut to the brain, due to the lack of a functioning BBB to prevent this leakage.

### 3.2. Invasion of the CNS and Role in Aβ Generation of AD-Associated Microorganisms and Viruses

Depending on the organism, there are several ways that pathogens can infiltrate the CNS and potentially further the progression of AD. The first is through a compromised BBB. Whereas a healthy and functional BBB normally provides a selective barrier to the passage of cells and molecules into the brain, a compromised BBB can allow direct entry into the cerebral spinal fluid via the bloodstream [21]. This places aging populations and those with weakened immune responses especially at risk, as some viruses, such as herpesvirus, can remain latent after initial infection and then reactivate in aging individuals long after, to introduce delayed adverse complications [71]. Even with a healthy BBB, however, bacteria and viruses are still able to be introduced into the brain through various mechanisms. HIV, for example, is carried from the immune system to the brain by infected leukocytes that are able to cross the BBB. *P.* gingivalis and other oral spirochetes have also been suggested to be capable of invading the CNS via the oral cavity, through the trigeminal nerves and ganglia [42]. Additionally, pathogens such as bacteria and viruses can bypass the BBB altogether by entering through the olfactory system, as the nasal cavity connects the peripheral environment to brain regions such as the olfactory bulb [72], the entorhinal cortex and the hippocampus, which traditionally receive smell sensory signals. *C. pneumoniae*, a respiratory pathogen, has specifically been suggested to enter the brain through the olfactory system, with its presence detected in the entorhinal cortex and hippocampal formation of AD patients [73]. A recent 2020 study model demonstrated that exposure to *C. pneumoniae* via the olfactory system was sufficient to induce Aβ plaque and NFT formation in the olfactory cortex and hippocampus of immunocompromised individuals [74]. This is further evidenced by Little et al., who found that intranasal inoculation of *C. pneumoniae* was sufficient to induce AD-like traits in mice [75]. Once in the brain, there are several ways in which these pathogens contribute to the Aβ production that is characteristic to AD. One mechanism is through the alteration of gene expression. The study previously mentioned by Readhead et al. found that HHV-6 and HHV-7 interact with known regulatory genes responsible for amyloid processing, such as the amyloid beta A4 precursor protein-binding family (*APBB2*), clusterin (*CLU*), and gamma-secretase subunit presenilin-1 (*PSEN1*) [61]. Similarly, infection of *C. pneumoniae* within human neuronal cell cultures possibly alters calcium-related gene expression such that they express patterns similar to those reported in AD brain samples [76]. Another way viruses can influence Aβ production is through protein misfolding. Specifically, viruses such as HHV, cytomegalovirus and Epstein Barr Virus have been shown to contain prion-like domains that may trigger the misfolding of proteins like Aβ [77]. Through these varying mechanisms, many microorganisms and viruses have been found to initiate Aβ plaque formation. 

In-vivo studies have noted a correlation between viral and bacterial infections and the accumulation of Aβ peptides. Mice infected with HSV-1 [78], pseudorabies virus [79], *C. pneumoniae* [75] and *P. gingivalis* [68,80] were found to have a significantly increased level of Aβ_1–42_ in the brain. In addition, Aβ expression was found to be upregulated in rats exposed to bacterial pathogens. HSV-1 has also been found to infect the hippocampus region at a greater rate; the same area found to have greater amounts of Aβ plaques in AD [81]. In vitro studies observed cells co-cultured with either HSV-1, HSV-2, *P. gingivalis* or *B. burgdoferi* to have increased intracellular concentrations of Aβ [80,82,83,84,85,86,87,88,89]. Furthermore, HSV-1 has been associated with the inhibition of the non-amyloidogenic pathway of APP metabolism, and the increased expression of β-secretase. This is evidenced by a 2011 study indicating the direct and frequent interaction between HSV-1 and AβPP [90]. Aβ plaques have also been identified in the brains of HIV-1-infected individuals. Autopsies performed on 162 HIV positive individuals found roughly half of them to contain Aβ plaques [91]. Cell culture studies observed an increase in Aβ production and secretion following exposure to mRNA and proteins from the HIV Nef gene [92].

It must be noted, however, that the association between infection and Aβ plaque formation is not consistent across all populations. For example, in the study previously mentioned by Sundar et al., younger and healthier individuals exposed to *C. pneumoniae* did not exhibit the same AB peptide and NFT formation as older and immunocompromised subjects [74]. Moreover, it has been observed that genetic discrepancies, especially in the APOE gene, influence one’s susceptibility to HSV-1 infection and subsequent AD development. Specifically, the APOEε4 allele places individuals at a greater risk of developing HSV-1-associated AD, with a combination of APOEε4 and HSV-1 comprising 60% of all AD cases [93,94]. This finding has been recapitulated in animal studies, where mice with the APOEε4 allele display a greater viral load than mice with other allele types after HSV-1 infection [95].

### 3.3. Aβ as an Antimicrobial Peptide

It is clear that a relation exists between bacterial and viral infections and Aβ production rate, as described in the previous section. Aβ peptides have long been thought to lack any physiological function; however, this notion has been challenged in recent years. Clinical studies have observed the depletion of Aβ peptides, through anti-Aβ therapies, to increase the rate of infections in some participants. Furthermore, Aβ plaques have been found to contain microbial and viral DNA, such as HSV-1. One study identified HSV-1 virus DNA in roughly 90% of Aβ plaques [96]. In addition, AD brains have been associated with 5- to 10-fold increases in bacterial read compared to control brains [64]. In the presence of bacterial lipopolysaccharides, microglial cells have also been shown to upregulate Aβ production [97]. From these and similar findings, it has been suggested that the pathogenesis of AD could be triggered by viral and/or microbial infections. These observations led to the recent development of the antimicrobial protection hypothesis for AD, which explores the notion of Aβ peptides having a role in innate immunity as an AMP that aids in the entrapment and degradation of invading bacteria and viruses.

The innate immune system utilizes AMPs to target invading microorganisms, such as bacteria, viruses, fungi, and in some instances cancerous cells. Mammalian AMPs exist in three main families: defensins, histatins and cathelicidins [98]. Similar to how Aβ peptides are generated through the two-step cleavage of APP, AMPs are also formed from the breakdown of larger precursor proteins. Examples of amyloid AMPs that have a role in immunity are present in the human body. Amyloidogenic major basic protein-1 (MBP-1) is implemented in eosinophils against pathogens [99]. Like Aβ peptides, MBP-1 also forms aggregates, specifically at the surface of the bacteria to limit its spread. Further support of Aβ peptides being AMP stems from their similarity to AMP LL-37, the only cathelicidin identified thus far in humans. Both compounds exhibit tendencies to form cytotoxic soluble oligomers and insoluble fibrils, characteristic features of tinctorial amyloid [98]. Additionally, deficiency in the latter can result in Kostmann syndrome, an immunodeficiency disorder that, if left untreated, can result in death due to infection within the first year of life [81]. High levels of LL-37 are likewise dangerous, as they has been associated with the development of plaques in atherosclerosis and other non-infectious diseases [98]. Protein analyses comparing known AMPs and Aβ peptides demonstrate structural similarities between these peptides, as well sequential similarities pointing to a shared homology between Aβ peptides and a specific family of bacteriocins [100]. This is particularly notable as bacteriocins are traditionally synthesized by bacteria as part of an antimicrobial response to contact with closely-related strains [101]. If verified as an AMP, Aβ would not be the only AMP suggested to be involved in AD. Often expressed in epithelial cells, β-defensin-1 is significantly elevated in astrocytes of the hippocampus, the choroid plexus, and the granulovacuolar degeneration structures of AD brain samples [102].

In vitro studies suggest the ability of Aβ peptides to be an AMP, and inhibit growth of a number of bacteria and viruses. In respect to the latter, Aβ has been shown to have antiviral activity against both HSV-1 and the influenza virus A by inhibiting the infectivity of HSV-1 [82], influenza virus A [103], H3N2 [103] and H1N1 [103]. Researchers found that in mouse and human neural cell cultures, Aβ peptide deposition was accelerated in response to HSV-1 and HHV6 infection, with the oligomers binding to the viruses as part of a protective entrapment mechanism [104]. Aβ peptides have also been shown to have antimicrobial properties against both Gram-positive and Gram-negative bacteria, including *Enterococcus faecalis*, *Escherichia coli*, *Listeria monocytogenes*, *Salmonella typhimurium*, *Staphylococcus aureus*, *Staphylococcus epidermidis*, *Streptococcus agalactiae* and *Streptococcus pneumoniae* [30]. In a study comparing Aβ peptides and LL-37, Soscia et al. found that, against eight different bacteria and viruses, Aβ peptides demonstrated an antimicrobial activity equivalent to, and sometimes even greater than, the known AMP LL-37. The same study also found that, when comparing the brain homogenates of Aβ-enriched areas from both AD and non-AD brains, the AD brain samples had elevated antimicrobial and antiviral activity. These discrepancies were eliminated once the AD brain tissues were immunodepleted using anti-Aβ antibodies. It is important to consider the sequence length of the Aβ peptide when examining its antimicrobial capabilities, as Aβ_1–42_ was shown to be capable of binding to the surface of bacteria and aggregating into clusters, whereas other peptide lengths were not [105].

Animal studies have further evidenced the important potential role that Aβ peptides have in protecting against infections. In a 2016 study, researchers tested Aβ’s functionality as an AMP in mice and nematode models [106]. Kumar et al. found that transgenic 5XFAD mice, which constitutively express human Aβ peptides, survived significantly longer than wild-type mice after the injection of *Salmonella typhimurium* into their brains. The 5XFAD mice were observed to have accelerated Aβ deposition that closely co-localized with the bacteria, reducing their cerebral viral load compared to wild-type mice. These findings were recapitulated again in worm models, as nematodes expressing Aβ were found to have increased survival following fungal infection of *C. albicans*, compared to nematodes that did not produce Aβ peptides. Additionally, it has been observed that impaired mice that lacked the ability to generate Aβ peptides have been shown to have increased postpartum mortality, which was only reversed by maintaining a sterile environment [107]. Furthermore, APP knockout mice were also observed to have increased rates of mortality. Altogether, these studies support the notion that Aβ peptides are an AMP, and an integral part of the brain’s innate immune response against invading pathogens.

**Figure 2:**
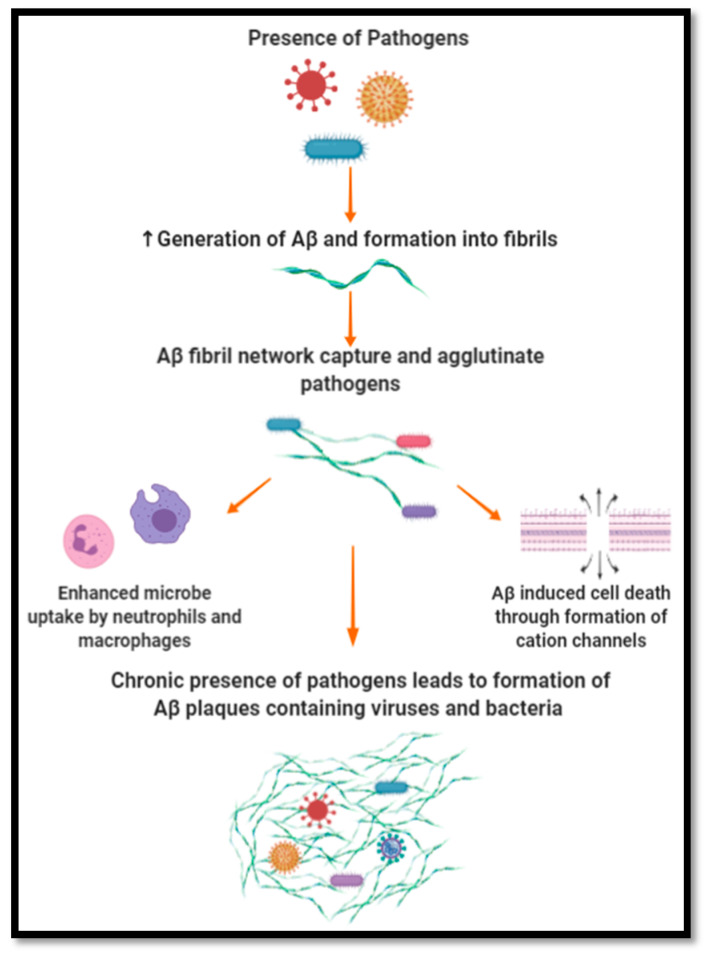
Aβ as an antimicrobial peptide.

The mechanism by which Aβ peptides have been suggested to exert their antimicrobial and antiviral effect has been based on entrapment and lytic activity, as illustrated in Figure 2. Being a self-complimentary peptide containing two distinct hydrophobic and hydrophilic surfaces, Aβ peptides can self-assemble into oligomers. As oligomerization continues, a fibril network is created which targets, captures and agglutinates microbes, limiting their proliferation and impact on their environment. Aβ peptide affinity towards microbes has been suggested to be due to its positive charge and the microbe’s negatively charged membrane [30,108,109]. Furthermore, the ability of Aβ peptides to agglutinate microbes stems from its heparin-binding activity, which is able to target carbohydrates present on the surface of microbes. Once entrapped, it is suggested that Aβ peptides induce cell membrane disruption by forming cation channels. These channels cause ion dyshomeostasis and subsequent cell death. This mechanism is similar to the activity observed in AMPs such as LL-37, which assert protection through microbial agglutination and entrapment. The oligomerization activity observed with Aβ peptides is a common trait of AMPs, which mediates their ability to entrap and lyse pathogens while maintaining their resistance to protease activity. The entrapment of microbes and viruses can also enhance their uptake by neutrophils and macrophages. In respect to HSV-1, Aβ peptides have been proposed to interfere with its ability to fuse with the plasma membrane of cells, hindering its infective ability [110].

## 4. Potential Novel Therapeutics

In aligning with the antimicrobial hypothesis of AD, the use of antimicrobial and antiviral therapeutics could prove to be effective in targeting the root cause of AD. It should be noted, however, that the chronic over-production of Aβ peptides, which form numerous insoluble plaques, would also need to be addressed. As such, the primary aim of these drugs would be to target bacteria and viruses, but a secondary aim would be to reduce the already-present Aβ burden that the brain is under.

### 4.1. Antiviral Drugs

#### 4.1.1. Acyclovir

Acyclovir is an antiviral drug that is used for HSV-1 infections, which has been found to be well tolerated and safe [111]. By inhibiting the virus’s replication, this antiviral agent is able to reduce the viral load exhibited by HSV-1. Administration of acyclovir in HSV-1-infected cells has been found to cause a significant reduction of HSV-1 proteins, along with a reduction of roughly 28% in Aβ accumulation, compared to untreated cells. The study associated this with reduced levels of β-secretase and a component of γ-secretase which metabolizes APP into Aβ [112]. Acyclovir administration has also shown to prevent HSV-1-related neuronal death [113]. In relation to the cognitive impairment observed in AD, a study by Hui et al. investigated the effects of acyclovir on Aβ oligomer-induced spatial cognitive impairments. The study found that the co-administration of acyclovir with dexamethasone attenuated impairments in spatial cognition. Furthermore, this combination reduced the levels of neuroinflammation markers such as TNF-α and IL-6, along with microglia activation. Interestingly, the study found these effects to only occur when acyclovir and dexamethasone were administered together [114].

#### 4.1.2. Penciclovir

Penciclovir is another antiviral drug that targets HSV-1 DNA replication by blocking chain elongation. Cell cultures infected with HSV-1 displayed a reduction of virus and Aβ accumulation when penciclovir was administered. This was paralleled with a reduction in β-secretase and a component of γ-secretase [112].

#### 4.1.3. Foscarnet

Foscarnet has been tested for its ability to reduce HSV-1 levels in vitro. A study found it was able to reduce Aβ accumulation, although only at higher doses. It was also unable to significantly reduce virus levels. Furthermore, foscarnet was not as effective as acyclovir or penciclovir, and hence currently is not seen as the optimal antiviral drug available for AD [112].

#### 4.1.4. Valacyclovir

Valacyclovir, an antiviral medication used in HSV-1 and HSV-2 infections, has been determined to positively impact cognition by improving visual object learning, verbal memory and working memory in patients with schizophrenia [115]. Due to its effects on working memory, its effectiveness against HSV-1 and HSV-2, and its generally safe consumption, valacyclovir has been suggested as a potential therapeutic for AD. A clinical study is currently underway in which patients that both have mild AD and tested positive for HSV-1 or HSV-2 will receive valacyclovir. The aim of the study is to determine the impact of this treatment on cognition and the accumulation of amyloid and tau [116].

#### 4.1.5. Bay 57-1293

Numerous studies have determined the antiviral agent Bay 57-1293 to be effective in combating HSV-1 [117,118,119,120,121]. By targeting the helicase–primase complex, Bay 57-1293 can inhibit viral DNA replication, and has been found to be more potent than acyclovir. The severity and frequency of recurring HSV was also found to be reduced by use of this drug [121]. Furthermore, it was able to decrease levels of Aβ and reduce P-tau production in Vero cells infected with HSV-1 [117].

#### 4.1.6. Biflavonoids

Our lab has investigated the use of bioflavonoids, including ginkgetin, isoginkgetin and ginkgolic acid, derived from the leaves of Ginkgo biloba. The antiviral capabilities of these compounds has been well established in previous studies [122,123,124,125,126]. Hayashi et al. determined ginkgetin to successfully inhibit the viral replication of HSV-1, HSV-2 and the human cytomegalovirus, while also suppressing viral protein synthesis [122]. Additionally, a study by Miki et al. found ginkgetin to have anti-influenza virus activity [123]. Ginkgetin has been studied for use in AD by Zeng et al., who administered the drug to APP/PS1 transgenic mice. They observed a significant reduction in Aβ plaques and an improvement in inflammation [127]. Borenstein et al. have demonstrated the ability of ginkgolic acid to limit virus infectivity by inhibiting its fusion. The study found ginkgolic acid to be successful in inhibiting HSV-1, human cytomegalovirus and zika virus. Furthermore, it was effective in inhibiting viral protein synthesis and genome replication, in HSV-1 and human cytomegalovirus, respectively [124]. Ginkgolic acid has also demonstrated antimicrobial properties, specifically against *E. coli* and *Staphlylococcus aureus* [125]. Isoginkgetin has been shown to provide neuroprotection against the cytotoxic effects of excessive Aβ accumulation [128,129], while also having anti-microbial and anti-fungal activity [126]. Our lab’s preliminary work in testing these three compounds in AD determined their effectiveness in reducing Aβ load in vitro, further supporting their therapeutic potential in AD.

### 4.2. Antimicrobial Drugs

#### 4.2.1. Doxycycline

Doxycycline is a tetracycline antibiotic that has been studied for its therapeutic efficacy in AD models. Contrary to other tetracyclines, doxycycline has been determined to be safe and is able to penetrate the BBB [130], allowing it to exert its effect directly in the CNS. In vivo models, in which doxycycline was administered to mice, observed its accumulation in amyloid deposits, including Aβ plaques [131]. With respect to the production and formation of Aβ oligomers, it was observed that although doxycycline administration in transgenic mice did not cause a shift in Aβ monomers, there was a significant reduction in Aβ 18-mer levels when compared to control [132]. The same study also observed a significant memory recovery in animals that received treatment; however, there was no reduction in Aβ plaque size [132]. The paper suggested this was possibly due to the short two-month period of the study, as a previous three-month study found plaque size to be significantly reduced [132]. In respect to neuroinflammation, a reduction in microglia activation has also been associated with doxycycline administration [132]. A drosophila model, which administered doxycycline to Aβ_1–42_-expressing flies, observed that the treated group’s locomotor deficits developed slower than the control group. The same study also observed doxycycline administration to be associated with reduced Aβ fibrilization, suggesting the production of smaller amyloid structures [133]. Another study associated doxycycline with the destabilization of Aβ fibrils [134]. Clinical trials, however, were not as successful. One study, which administered doxycycline and rifampicin, observed improvements in cognitive function, as assessed by the Standardized Alzheimer’s Disease Assessment Scale–Cognitive Subscale (SADAScog) score [135]. However, a second study did not find any improvements in the cognition or function of patients with mild to moderate AD with doxycycline/rifampicin administration [136]. Further investigations would be needed to understand why the benefits seen in murine models do not translate into clinical trials.

#### 4.2.2. Propranolol

Propranolol hydrochloride, an antihypertensive drug shown to have antimicrobial properties [137], has also been found to impact Aβ production. Cortico-hippocampal neuronal cultures treated with this drug manifested reduced levels of Aβ production. Furthermore, the one-month treatment of Tg2576 mice resulted in roughly a 40% reduction of Aβ_1–40_ and Aβ_1–42_ levels in the brain. When administered over a period of 6 months, Aβ peptide levels were still reduced in the brain; however, no improvement in spatial memory function was observed [138].

#### 4.2.3. Rifampicin

Rifampicin is an antibiotic derived from *Nocardia mediterranei*, which has been investigated for use in neurodegenerative diseases such as Parkinson’s and AD [139,140]. Rifampicin has been found to provide neuroprotection through its anti-oxidant and anti-inflammatory properties [139,141]. Furthermore, in vitro studies found that its administration improved neuronal survival and reduced microglial activation [141]. Studies by Tomiyama et al. found rifampicin to protect neurons from cytotoxicity by scavenging free radicals [142,143]. In relation to the antimicrobial hypothesis, rifampicin has been previously studied for use in bacterial cerebral infections [144]. As rifampicin is able to cross the BBB [144], it can exert its antimicrobial effect directly in the brain. In the presence of rifampicin, a reduction of Aβ fibril formation [142] has been observed in addition to augmented Aβ clearance [145]. A study by Umeda et al., in which rifampicin was administered to APPOSK mice, found the treatment to reduce Aβ accumulation, provide synaptic protection, and reduce microglial activation [146]. Clinical studies exploring the impact of rifampicin on cognitive function have also been investigated, as mentioned in previous sections. Even with its many benefits, the oral intake of rifampicin has also been associated with liver injury in humans. To circumvent this limitation, administering rifampicin intranasally or subcutaneously has been suggested [147]. These routes of rifampicin administration have been shown to be more effective in improving memory than oral administration [147].

#### 4.2.4. Gingipain Inhibitors

The use of gingipain inhibitors in AD is another approach that has been taken to alleviate the negative impact of the disease. Gingipains are virulence factors that are produced by *P. gingivalis* [148]. They are made up of a group of cysteine proteinases, specifically arginin–gingipain A, arginine–gingipain B, and lysign–gingipain [148,149]. Given the key role gingipains play in host colonization [148] and the inactivation of host defenses [150,151,152], they are essential for the survival and pathogenicity of *P. gingivalis*. Regarding Aβ_1–42_ peptide production, *P. gingivalis* infection was found to increase Aβ_1–42_ levels. Furthermore, incubating *P. gingivalis* with Aβ_1–42_ peptides led to a significant increase in *P. gingivalis* death. These two findings further support the antimicrobial hypothesis for Aβ peptides [153]. Gingipain inhibitors, such as COR286, COR271 and COR388, have been found to be effective in inducing *P. gingivalis* death and reducing the bacterial load in the brain, more so than other antibiotics, such as moxifloxacin [153,154]. In addition, COR271 was found to provide some level of neuroprotection as well [153]. The administration of gingipain inhibitors has also been associated with a decrease in host Aβ_1–42_ response to *P. gingivalis* infection [68].

### 4.3. Limitations

Even with the benefits associated with the antimicrobial and antiviral drugs listed above, insights into their mechanism of action and their impact on Aβ peptide levels are needed. A greater understanding as to whether their administration indirectly reduces the presence of Aβ peptides by reducing the viral/bacterial load on the brain, or if they act directly in reducing Aβ peptides level, is needed. If it is the latter, and the antimicrobial hypothesis for Aβ peptides holds true, their efficacy might not be as positive as hoped. In addition, it is important that the chosen antimicrobial or antiviral drug does not have any adverse effects that could take away from its benefits. For example, cefepime is an antibiotic that has shown to be able to cross the blood-brain barrier and cause neurotoxic symptoms [154].

## 5. Conclusions and Future Directions

The findings of the numerous studies highlighted in this review present a clear indication of the role bacteria and viruses can have in AD development. Even with this conclusion, it is clear that a specific bacteria or virus alone is not responsible for AD development, as no specific bacteria or virus has been identified to be universally present in all AD brains. Rather, a number of viruses and bacteria could exacerbate the progression of neurodegenerative diseases, either independently or along with other pathogens. By exploring the presence of multiple viruses/bacteria in AD brains, future investigations can give insights into which microorganisms are most present, and whether all AD brains have both a detected and increased level of selected bacteria/virus. The use of antiviral and antimicrobial drugs early on, while the patient is still in the presymptomatic phase of AD, could have potential effectiveness in targeting the root cause of AD pathogenesis and alleviating the viral/microbial load on the brain. Further investigations into their use in AD would give greater insight regarding their efficacy and limitations.

## Abbreviations

AD	Alzheimer’s Disease
Aβ	amyloid-β
AMP	antimicrobial peptide
APP	amyloid precursor proteins
BACE1	B-site ABPP cleaving enzyme
BBB	blood brain barrier
CNS	central nervous system
HSV-1	herpes simplex virus-1
MBP-1	major basic protein-1
NFTS	neurofibrillary tangles
